# Documentation of Extended Focused Assessment with Sonography in Trauma (eFAST) Is Frequently Incomplete: A Prospective Observational Study

**DOI:** 10.5811/westjem.52905

**Published:** 2026-05-14

**Authors:** Magdelyn Feuerherdt, Miriam R. Elman, Bryson Hicks, Alfredo Sabbaj, William J. McLean, Aishwarya Sreenivasan, Cynthia Gregory, Kenton Gregory, Nikolai Schnittke

**Affiliations:** * Oregon Health & Science University, Center for Regenerative Medicine, Portland, Oregon; † Oregon Health & Sciences University, School of Public Health, Portland, Oregon; ‡ Oregon Health & Science University, Department of Emergency Medicine, Portland, Oregon; § Oregon Health & Science University, School of Medicine, Portland, Oregon; || Portland State University, School of Public Health, Portland, Oregon

## Abstract

**Introduction:**

The extended focused assessment with sonography in trauma (eFAST) is a point-of-care ultrasound protocol that identifies life-threatening thoracoabdominal trauma. Clinical documentation of the eFAST is essential to convey medical decisions, but the extent of documentation in clinical practice is unknown. This study describes the proportion of eFAST exams that are documented in the medical record, the type of documentation (free text vs procedure note), and clinical factors associated with documentation.

**Methods:**

This prospective, single-center study evaluated the documentation of consecutive eFAST exams performed at a single Level I trauma center between November 2021–November 2022. Research coordinators observed all trauma activations and noted whether any portion of an eFAST was performed. Our primary outcome was the presence of any documentation in the chart. We analyzed secondary outcomes using a multivariable logistic regression model and included the type of documentation (any documentation vs billable procedure note) as well as patient- (body mass index, age, sex, shock index > 1, and presence of pathology on reference test) and operator-level (involvement of ultrasound faculty) factors associated with each type of documentation.

**Results:**

A total of 335 patients had a witnessed eFAST performed during the study period. No documentation was observed in 114/335 (34%) patients compared to any documentation in 221/335 (66%). Most documentation was free text only in 134/335 (40%) patients, with only 87/335 (25.9%) of patients with billable documentation. Regression analysis found that shock index > 1 (adjusted odds ratio [aOR] 7.37; 95% CI, 2.55–31.29), presence of pathology on eFAST (aOR 2.07; 95% CI, 1.24–3.51), and involvement of ultrasound section faculty (aOR 1.93; 95% CI, 1.05–3.66) were significantly associated with an increase in any documentation.

**Conclusion:**

Approximately one-third of performed trauma eFAST exams are undocumented, and only one-quarter of exams have structured documentation that enables billing. Further work is needed to understand factors that can lead to improved documentation quality to communicate results, justify medical decision-making, and augment reimbursement.

## INTRODUCTION

The extended focused assessment with sonography in trauma (eFAST) is a point-of-care ultrasound (POCUS) protocol that enables rapid identification of life-threatening thoracoabdominal injuries, including hemoperitoneum, hemopericardium, hemothorax, and pneumothorax.^1^ Results of the eFAST guide emergent management such as tube thoracostomy placement, operative intervention, and/or blood product administration.^2^ Multiple organizations, including the American College of Emergency Physicians, the Centers for Medicare & Medicaid Services, and the American College of Surgeons, recommend standardized documentation of POCUS. These guidelines recommend including information on patient demographics, indications, views, findings, and interpretations in a structured procedure note. This documented information is used to communicate medical decision-making to other clinicians, billing/coding groups, and ultrasound faculty who provide education and feedback for their clinical groups.^3^–^5^

Despite the importance of documentation as a key element of patient care, many POCUS procedures may be documented only partially or not at all.^6^–^10^ There is little published evidence of specific barriers to documentation. A single, before-after retrospective study did reveal a positive effect of clinician education on documentation compliance, suggesting that clinicians may not receive enough guidance on documentation protocols, are unaware of the importance of documentation elements, or lack the confidence to document a scan as a billable procedure.^11^ Other potential barriers include the time-sensitive nature of trauma resuscitation and institutional lack of standardized, accessible documentation pathways. This gap in patient records, referred to as “phantom scans” in the literature,^9^ can have consequences for both patients and clinicians. Delayed communication of pertinent clinical information can reduce effective communication between clinicians. Inadequate documentation also impedes quality review and education efforts by ultrasound faculty who look at documented POCUS interpretation to identify operator knowledge gaps.

Incomplete POCUS documentation is difficult to study, because it is challenging to identify how many exams are performed if they remain undocumented.^9^ A lack of research on the topic means that the frequency of improper documentation and contributing factors is largely unknown. In this study, we took advantage of the presence of clinical research coordinators (CRC) at all trauma resuscitations. The CRCs tracked performance of the eFAST in a prospective manner. Our goal in this study was to describe the incidence of documentation of eFAST in trauma patients and to determine the impact of patient- and operator-level variables on documentation.

## METHODS

### Study Design and Setting

This single-center, prospective study took place at a Level I trauma center in the Portland, Oregon, metropolitan area between November 5, 2021–November 5, 2022, with an annual volume of 40,000 including 2,600 trauma patients. The institution is affiliated with a medical school as well as emergency medicine (EM) and general surgery residency programs. All trauma activations are attended by at least two trauma residents (a trauma chief resident and a junior resident) and an EM attending. The emergency department is equipped with a variety of ultrasound machines (Mindray TE7 and M9 platforms and Philips Lumify) with curvilinear, linear, and phased array transducers. The choice of machine, transducer, and preset was deferred to the clinicians performing the exam based on availability and clinical scenario. An EM resident is present at most trauma activations except during excused educational conference one morning a week. All EM attendings have hospital privileges to perform and interpret the eFAST. All incoming EM residents receive eight hours of training by ultrasound faculty in POCUS (including eFAST) prior to starting shifts followed by a four-week dedicated POCUS rotation during the first year of residency. All saved POCUS studies are backed up to the image repository ExoWorks (Exo Imaging, Inc, Santa Clara, CA) where members of the ultrasound section (four full-time faculty and one emergency ultrasound fellow) perform weekly quality assurance for targeted minimum of 80% of all uploaded studies.

**Population Health Research Capsule**

What do we already know about this issue?

*Past studies reported that point-of-care ultrasound exams are often not saved; however, the proportion of exams documented in the chart is unknown*.

What was the research question?

*We determined the proportion of performed extended focused assessment with sonography in trauma (eFAST) exams docu[1]mented in the health record*.

What was the major finding of the study?

*34% of trauma eFAST exams are not documented, and only 25.9% use structured documentation*.

How does this improve population health?

*Effective communication is essential to ensure appropriate care. By defining the problem of sparse eFAST documentation we identify a quality improvement goal*.

Patients were identified as part of a larger project to acquire ultrasound imaging for training artificial intelligence image guidance and interpretation algorithms. Trauma patients were screened for enrollment in the larger study based on whether a clinical ultrasound exam was performed. This study describes an analysis of the screening log, conducted under a waiver of informed consent approved by the institutional review board. The reporting of the study follows the Strengthening the Reporting of Observational Studies in Epidemiology (STROBE)^12^ and Standards for Point-of-care Ultrasound Research Reporting (SPUR)^13^ guidelines (Supplemental File).

### Patient Population

All consecutive trauma activations were attended by at least one CRC who observed whether any portion of the eFAST was performed (defined as observation of an ultrasound of the thorax and/or abdomen). Adult patients (≥ 18 years of age) who had an observed eFAST performed were entered into a screening log for the parent study and included in the analysis. We excluded pediatric patients and patients who did not have an eFAST performed. Due to the descriptive nature of the study, we did not perform a power calculation.

### Chart Review

Two trained, independent reviewers, blinded to the study goals performed chart review of the electronic health record (EHR) (Epic Systems Corporation, Verona, WI), following a standardized data collection guide (Supplemental File), and entered data in the REDCap electronic data capture tools hosted at Oregon Health & Sciences University.^14^ Collected data included patient demographics (sex, age, and body mass index [BMI] calculated as kilograms per square meter), vital signs (systolic and diastolic blood pressure, heart rate, calculated shock index), trauma mechanism (blunt or penetrating), surgical findings, computed tomography (CT) and findings, eFAST exam elements and findings, and the level of documentation present in the patient’s medical record. The involvement of an ultrasound faculty member was defined based on whether the author or co-signer of the note was ultrasound-fellowship trained or an ultrasound fellow during the study period. Additional data on the operator(s) involved (eg, medical student, EM resident, or surgery resident) was not reliably available in our dataset. Each pathologic element (hemoperitoneum, hemopericardium, pneumothorax, hemothorax) of the eFAST was considered assessed if it was explicitly mentioned in the documentation.

If free-text documentation did not explicitly address a pathology, we considered mention of a positive/negative FAST as assessing for hemoperitoneum and hemopericardium only, while mention of a positive/negative eFAST exam also assessed for thoracic pathology (pneumothorax and hemothorax). To test chart review accuracy, both reviewers abstracted 10% of the charts, and agreement was found to be high (all field kappa = 0.90 [95% CI, 0.84–0.96] and kappa for the primary documentation outcome = 0.90 [0.71–1.0]).

### Outcomes

The primary outcome was whether any eFAST documentation was found in the EHR notes (“any documentation”). The secondary outcome further categorized whether the documentation followed a structured procedure note, which is used at our institution for billing and coding (“billing documentation”). Both outcomes were defined dichotomously as having that class of documentation or not having it. The study focused on textual documentation and did not assess whether images were saved.

### Statistical Analysis

We reported patient demographic and clinical characteristics as frequency with percentage for categorical variables and median with interquartile range (IQR) for continuous variables. The presence of ultrasound documentation of patients with and without the reference standard (defined as pathology on CT or surgical confirmation) were compared by chi-square or Fisher exact tests, as appropriate.

We used multivariable logistic regression to determine the association between patient and clinical characteristics and the presence of eFAST documentation for both outcomes. Clinical knowledge was used to a priori select the set of characteristics to include in models for both outcomes: patient sex (female, male); BMI; calculated shock index (≤ 1, > 1); ultrasound faculty performed or supervised the eFAST (yes, no); and presence of confirmatory findings on CT (yes, no). Sixteen individuals had missing BMI values, and these subjects were excluded from regression models. Odds ratios (OR) from univariable and multivariable models were reported with 95% confidence intervals. Further details of the methods and model diagnostics are presented in the Supplemental File.

#### Sensitivity Analyses

In cases where data lack adequate case numbers for combinations of independent variables and outcome levels, estimates based on maximum likelihood estimation like from logistic regression may suffer from sparse data bias, which can cause overestimation.^15^ To assess the magnitude of sparse data bias in models for our primary outcome, we performed sensitivity analyses using a Bayesian approach to reduce this bias in logistic models (detail in Supplemental File). We used two-sided *P* values < 0.05 to assess statistical significance, and R software v4.3.1 (R Foundation for Statistical Computing, Vienna, Austria) for analysis.

## RESULTS

**Figure 1:**
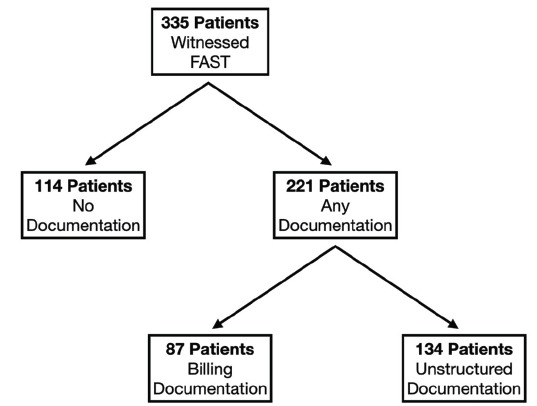
Flow diagram of documentation of extended focused assessment with sonography in trauma for patients with a witnessed ultrasound during trauma resuscitation. “No documentation” indicates patients with no reference to the eFAST in the medical record. “Any documentation” indicates patients with a reference to the eFAST in the medical record. “Billing documentation” indicates patients with a structured procedure note in the medical record. “Unstructured documentation” indicates patients with unstructured (free text) reference to the eFAST in the medical record. *eFAST*, extended focused assessment with sonography in trauma.

We identified 335 patients who had a witnessed ultrasound performed during their trauma evaluation (73.1% male, 26.9% female), with a median age of 40 years (IQR 29–58). Table 1 summarizes demographic features, characteristics of clinical presentation, and findings on confirmatory reference standard. Figure 1 demonstrates the frequency of no documentation (114/335, 34%), any documentation (221/335, 66%), billing documentation (87/335, 26%), and unstructured (free-text) documentation (134/335, 40%). Table 2 reports the elements of the eFAST and frequency of documentation based on pathology on reference standard (CT or surgical confirmatory test). Most documented ultrasounds reported views of the peritoneum (192, 57.3%) and pericardium (178, 53.1%), while a minority reported views of the lungs to evaluate for pneumothorax (106, 31.6%) and hemothorax (77, 23.0%). Patients with pathology on reference standard were significantly more likely to have any documentation for assessment of hemoperitoneum than those without pathology (76.7% vs 51.9%, *P* < .001), as were those with assessment of pneumothorax (52.9% vs 26.2%, *P* < .001).

Almost two-thirds of patients (221/335, 66%) had some form of documentation from the witnessed eFAST in their chart, while just over a quarter had complete billing documentation (87/335, 25.9%). Table 3 reports the patient characteristics by documentation class. In multivariable models of any documentation, shock index > 1 (adjusted OR [aOR]7.37; 95% CI, 2.55–31.29), positive reference standard (aOR: 2.07 [1.24–3.51]), and involvement of an ultrasound faculty operator (aOR 1.93, 1.05–3.66) were significantly associated with presence of any documentation (Table 4). By contrast, only the involvement of an ultrasound faculty operator was associated with the presence of billing documentation (aOR 3.72; 95% CI: 2.08–6.69).

In modeling the primary outcome (“any documentation”), the number of patients with a SI > 1 was rare among patients with no documentation (n = 4, Table 2). To assess overestimation from sparse data bias, we performed a Bayesian sensitivity analysis. This model suggests that SI retained a strong, significant association with any documentation even with models that shrink coefficients to address sparse data biases (Table S1).

## DISCUSSION

Standardized and accessible documentation is important for patients and clinicians. It allows for effective communication of a patient’s medical care, ensures continuity of care, and justifies medical decision-making. Documentation of POCUS findings is particularly important because operator skill plays an important role in image quality and interpretation. Deficiencies in real-world documentation of POCUS studies have been described but are difficult to study due to challenges in case identification. To the best of our knowledge, this is the first study to prospectively identify all eFAST studies that were performed during trauma activation. In this study, over one-third of patients who had a witnessed eFAST did not have the procedure documented, and only 25.9% had structured documentation that could be used for billing purposes.

Our results are consistent with other studies that have investigated phantom scanning, defined by a recent consensus statement as a POCUS “study that has not been documented in the medical record or has archived images; when audited it does not exist.”^9^ One single-center study evaluated the prevalence of unsaved images in trauma and cardiac arrest studies that were documented in the nursing run sheet and found that 86.5% of all studies had no corresponding saved images.^7^ A recent follow-up multicenter study by some of the same authors found substantial site variability with unsaved images found in 21.4–93.2% of eFAST exams, indicating heterogeneity in practice.^10^ Another retrospective study examined the documentation accuracy of eFAST with saved images and found that 29.8% (1,450/4,860) were undocumented, technically limited, or incomplete.^6^

To address these gaps in documentation a recent retrospective study from Sydney, Australia, found that implementation and teaching of an eFAST documentation guideline improved compliance during a three-month follow-up period.^11^ However, this was a relatively small study with a short follow-up period. More work is needed to understand the long-term impact of guideline education, as documentation guidelines have existed in the United States since at least 2001,^16^ yet compliance continues to be sparse. Taken together the existing evidence demonstrates a significant knowledge gap in defining the problem of phantom scanning and addressing factors contributing to this phenomenon.

To address factors that may contribute to documentation quality we focused on patient and operator characteristics. For patient characteristics we selected the presenting SI as a surrogate measure of illness severity because this metric is relatively simple to obtain and reduced drop-out due to missing data. The SI is also more directly related to severity of hemorrhagic or obstructive shock that is assessed by the eFAST than level of trauma activation or Injury Severity Score, which can be confounded by other factors such as brain injury.^17^,^18^ A SI > 1 was strongly associated with the presence of some documentation, but not with structured billing documentation. This may be due to the perceived need to document clinically significant findings but difficulty saving images and task saturation in sicker patients. The presence of pathology on confirmatory testing was also associated with increased documentation. Taken together, our findings suggest that clinicians are more likely to document positive findings in sicker patients who have a higher rate of acute interventions necessitating communication for continuity of care by multiple care teams.

Our results demonstrated a significant discrepancy in quality of documentation. Unstructured documentation was the most common but lacked details of the views acquired, interpretation by pathology, and indication for the exam. The eFAST assesses four different pathologies, but in unstructured documentation it is often listed simply as a binary (“FAST positive” or “FAST negative”). This lack of detail may explain why we observed less documentation of thoracic findings (pneumothorax and hemothorax), since documentation of a FAST as a binary exam does not include those thoracic views. In addition to billing, structured documentation facilitates consistent communication of relevant results and study limitations. Knowledge of the variables provided by structured documentation is essential for asynchronous review and feedback to identify opportunities for clinical operator improvement. At our institution, this image review is performed by members of the ultrasound faculty. Therefore, it is not surprising that involvement of these clinicians is most associated with structured billing documentation.

Future studies should focus on three features of phantom scanning: 1) qualitative study to understand barriers to documentation and help identify opportunities for improvement; 2) studies focusing on patient-centered consequences (eg, delays in care and appropriateness of care) to help define the impact of incomplete documentation and the need for documentation in clinician education; and 3) investigation into methods of improving documentation (such as workflow improvements and integration of machine-learning algorithms to automate saving and documenting the performed exams) to facilitate better care of injured patients.

## LIMITATIONS

This was a single-center study, which limits external validity; therefore, the results may not be generalizable to other settings, particularly non-academic trauma centers. To account for this limitation we provide a detailed description of our setting in the methods. We suspect that our data are a “best case” estimate of eFAST documentation, given the POCUS infrastructure at our institution. In addition, while we used a novel, consecutive-case identification strategy, it is possible that the CRCs missed a POCUS being performed, particularly during more chaotic resuscitations. An analysis of video recordings of the resuscitation (which our institution initiated after the study period) may provide a more accurate assessment of the true number of ultrasounds performed.

The study was also limited by the selected classes of documentation, which assumes that documentation in each class (any documentation and billing documentation) is uniform and that the same level of documentation is indicated for all eFAST exams performed. In fact, documentation is variable even within these classes (eg, some free-text documentation may include substantial detail, while some structured documentation may be missing important elements). Additionally, we were unable to assess why a clinician chose to document in a certain way. It is possible that some studies were performed for educational purposes and were not felt to be clinically indicated. To account for this confounder, we only included the initial evaluation when the CRC was present to witness the clinical care. During this phase of resuscitation there are fewer educational scans performed as these studies are typically deferred for more stable patients after their return from CT. It is likely that additional heterogeneity of clinicians (EM vs surgery resident or faculty) further contributed to documentation compliance; however, we were unable to reliably identify the clinician performing the ultrasound within the limitations of our data set.

Finally, we did not consider whether ultrasound images were saved. Saved images are a prerequisite for billing, and it is possible that the operator did not complete billing documentation knowing that they were unable to save images. This is an inherent limitation of POCUS, especially during task-saturated resuscitations of sicker patients. However, complete documentation is particularly important in these patients as the ultrasound results are most likely to guide the operator toward critical, high-stakes interventions.

## CONCLUSION

This prospective study of documentation of extended focused assessment with sonography for trauma during trauma resuscitations describes deficiencies in an existing documentation workflow. Severity of illness and the presence of pathology are associated with an increase in documentation. Involvement of an ultrasound faculty member was associated with documentation of eFAST findings in a structured, billable format.

## Supplementary Information



## Figures and Tables

**Table 1:** Demographics and clinical characteristics of patients with a witnessed extended focused assessment with sonography for trauma in a study of the frequency of documentation of these ultrasound exams.

Characteristic	Study patients (N = 335)
Sex, n (%)	
Male	244 (73.1)
Female	90 (26.9)
Age, median (IQR)	40.0 (29.0, 58.0)
BMI, median (IQR)	27.0 (23.6, 30.7)
Missing, n (%)	16 (4.8)
Heart rate, median (IQR)	95.0 (80.0, 111.0)
Shock index ≤ 1, n (%)	290 (86.6)
Pulse pressure, median (IQR)	43.0 (29.0, 55.0)
Mechanism of injury, n (%)	
Blunt	275 (82.1)
Penetrating	57 (17.0)
Missing	3 (0.9)
CT performed, n (%)	307 (91.6)
Surgery performed, n (%)	53 (15.8)
Pathology identified on reference standard, n (%)^a^	
Hemoperitoneum	73 (21.8)
Hemopericardium	8 (2.4)
Pneumothorax	68 (20.3)
Hemothorax	45 (13.4)

a Reference standard includes CT findings, surgical findings, and/or procedural findings such as chest tube placement.

*BMI*, body mass index; *CT, c*omputed tomography*; eFAST*, extended focused assessment with sonography in trauma; *IQR*, interquartile range.

**Table 2:** Assessment of documentation from study patients based on elements of the extended focused assessment with sonography in trauma and presence of pathology on reference standard (CT or surgical confirmatory test).^a^

POCUS documentation (n, % of total 335 patients)	Pathology on reference standard		P^b^
Hemoperitoneum Assessed (192, 57.3)	Yes (n = 73)	No (n = 262)	< .001
Any Documentation	56 (76.7)	136 (51.9)	
No Documentation	17 (23.3)	126 (48.1)	
Hemopericardium Assessed (178, 53.1)	Yes (n = 8)	No (n = 327)	.29
Any Documentation	6 (75.0)	172 (52.6)	
No Documentation	2 (25.0)	155 (47.4)	
Pneumothorax Assessed (106, 31.6)	Yes (n = 68)	No (n = 267)	< .001
Any Documentation	36 (52.9)	70 (26.2)	
No Documentation	32 (47.1)	197 (73.8)	
Hemothorax Assessed (77, 23.0)	Yes (n = 45)	No (n = 290)	.41
Any Documentation	13 (28.9)	64 (22.1)	
No Documentation	32 (71.1)	226 (77.9)	

a The number of patients (% of total, N = 335) is reported for the elements of the eFast with pathology present or absent on reference standard.

b Fisher exact test used for hemopericardium; all others are chi-square test.

*CT, c*omputed tomography; *eFAST*, extended focused assessment with sonography in trauma; *POCUS*, point-of-care ultrasound.

**Table 3:** Patient demographic and clinical characteristics of patients with a witnessed extended focused assessment for trauma by documentation class.

Characteristic	Any documentation		Billing documentation	
	
	Yes (n = 221, 66.0%)	No (n = 114, 34.0%)	Yes (n = 87, 26.0%)	No (n = 248, 74.0%)
Sex, n (%)				
Male	160 (72.4)	85 (74.6)	65 (74.7)	180 (72.6)
Female	61 (27.6)	29 (25.4)	22 (25.3)	68 (27.4)
Shock index, n (%)				
≤1	180 (81.4)	110 (96.5)	74 (85.1)	216 (87.1)
>1	41 (18.6)	4 (3.5)	13 (14.9)	32 (12.9)
BMI, median (IQR)	26.7 (23.5, 30.8)	27.2 (23.8, 30.6)	26.7 (22.6, 33.5)	27.1 (23.8, 30.5)
Missing, n (%)	10 (4.5)	6 (5.3)	4 (4.6)	12 (3.6)
Ultrasound faculty operator, n (%)	53 (24.0)	19 (16.7)	33 (37.9)	39 (15.7)
Positive reference standard, n (%)	101 (45.7)	31 (27.2)	39 (44.8)	93 (37.5)

*BMI*, body mass index; *eFAST*, extended focused assessment with sonography in trauma; *IQR*, interquartile range.

**Table 4:** Results of logistic regression for documentation outcomes (n = 319 patients).^*^

	Any documentation				Billing documentation			
	
	Univariable Models		Multivariable Model		Univariable Models		Multivariable Model	
Variable	OR (95% CI)	*P*	aOR (95% CI)	*P*	OR (95% CI)	*P*	aOR (95% CI)	*P*
SI > 1	7.94 (2.79, 33.40)	< .001	7.37 (2.55, 31.29)	.001	1.33 (0.63, 2.64)	.44	1.34 (0.62, 2.78)	.45
Positive reference	2.15 (1.32, 3.58)	.003	2.07 (1.24, 3.51)	0.01	1.35 (0.81, 2.23)	.25	1.58 (0.92, 2.72)	.10
US faculty operator	1.59 (0.89, 2.96)	.13	1.93 (1.05, 3.66)	0.04	3.37 (1.92, 5.95)	< .001	3.72 (2.08, 6.69)	< .001
Female	1.16 (0.69, 2.00)	.57	1.18 (0.68, 2.07)	0.56	0.97 (0.54, 1.69)	.91	0.96 (0.53, 1.71)	.89
BMI	1.00 (0.97, 1.04)	.86	1.01 (0.97, 1.05)	0.63	1.02 (0.98, 1.05)	.33	1.02 (0.98, 1.06)	.29

16 patients with missing BMI were excluded from the regression.

*BMI, b*ody mass index *OR*, odds ratio; *aOR*, adjusted odds ratio; *SI*, shock index; *US*, ultrasound.
