# Supplementary material for: Documentation of Extended Focused Assessment with Sonography in Trauma (eFAST) Is Frequently Incomplete: A Prospective Observational Study
Source: West J Emerg Med. 2026 May 14;27(3):629–35. doi: 10.5811/westjem.52905 (PMC13246171; doi:10.5811/westjem.52905)
Supplement: Supplementary file 1 [file wjem-27-629-s001.docx]

SUPPLEMENTAL MATERIAL

**Documentation of the eFAST Is Frequently Incomplete: A Prospective Observational Study.**

**Contents:**

1. Detail of regression model diagnostics (pages 2-17)
2. Detail of methods used for sensitivity analyses (pages 18-20)
   1. Supplemental Table 1
3. Reporting Guideline Checklists (pages 21-26)
   1. SPUR – Standards for Point-of-care Ultrasound Research Reporting
   2. STROBE - STrengthening the Reporting of OBservational studies in Epidemiology.
4. Data Collection Guide (Separate File)
5. **Detail of regression model diagnostics**

We followed traditional model diagnostics for assessing logistic regression (Zhang 2016), supplemented by a simulation-based approach that is designed to address the challenges of interpreting model diagnostics for logistic regression (Dunn and Smyth 1996, Gelman and Hill 2007). Plots for Pearson residuals versus fitted values and model variables were examined to assess model fit, appropriateness of the logit link, and functional form of continuous variables. Influence diagnostics were evaluated using Cook’s distance and leverage values; the presence of outliers was assessed with studentized residuals. We also generated standardized residuals using the *DHARMa* package in R, which produces scaled residuals that can be interpreted analogously to residuals from linear regression models (Hartig 2024). For this purpose, we simulated 1,000 residuals using the DHARMa simulateResiduals() function, which is recommended by the author for stable values.

Model calibration was evaluated graphically using calibration curves and quantitatively using average (Eavg) and maximum (Emax) absolute calibration error, which measure the mean and worst-case differences between predicted and observed probabilities. Overall predictive accuracy was assessed using the rescaled Brier score, and discrimination was summarized by the C-statistic. The 95% confidence intervals for these measures were calculated using 1,000 bootstrap samples.

Multicollinearity was evaluated using variance inflation factors (VIFs) for predictors included in the model.

**Model diagnostics: Any documentation**

**Figure A1.** Plots of Pearson residuals versus predictors and predicted values


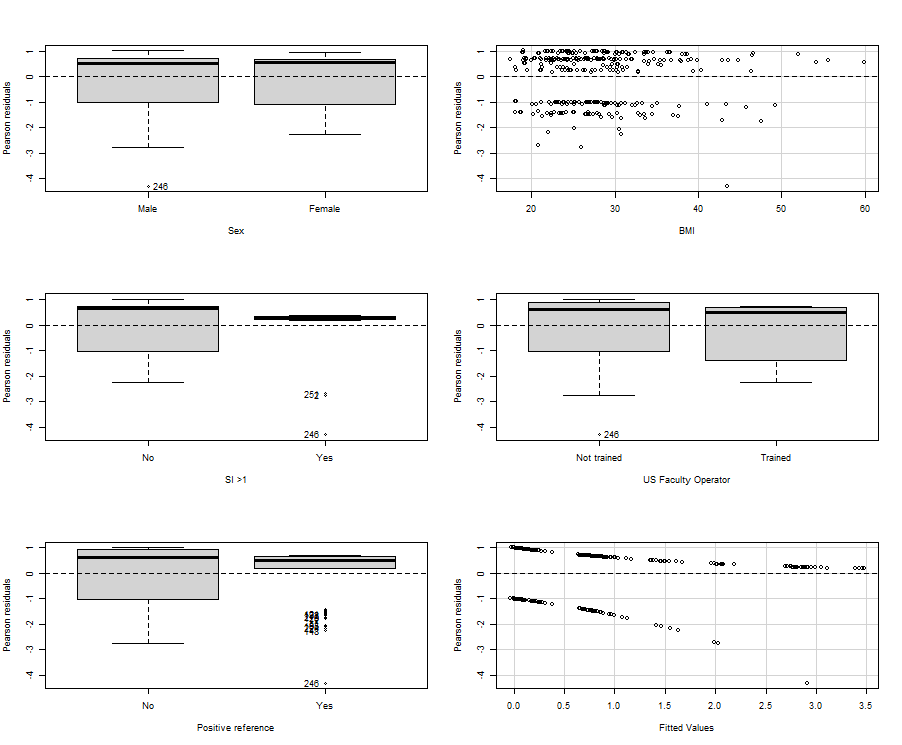


Figure A1 displays Pearson’s residuals plotted against individual predictors and fitted values. These plots indicate no obvious systematic departure from model assumptions. Residual patterns are consistent with the discrete nature of the binary outcome and show no clear evidence of nonlinearity or overall lack of fit. Additional diagnostics based on simulation-based residual analyses were used to further assess model adequacy.

**Figure A2.** Plot of studentized residuals vs fitted values to identify potential outliers


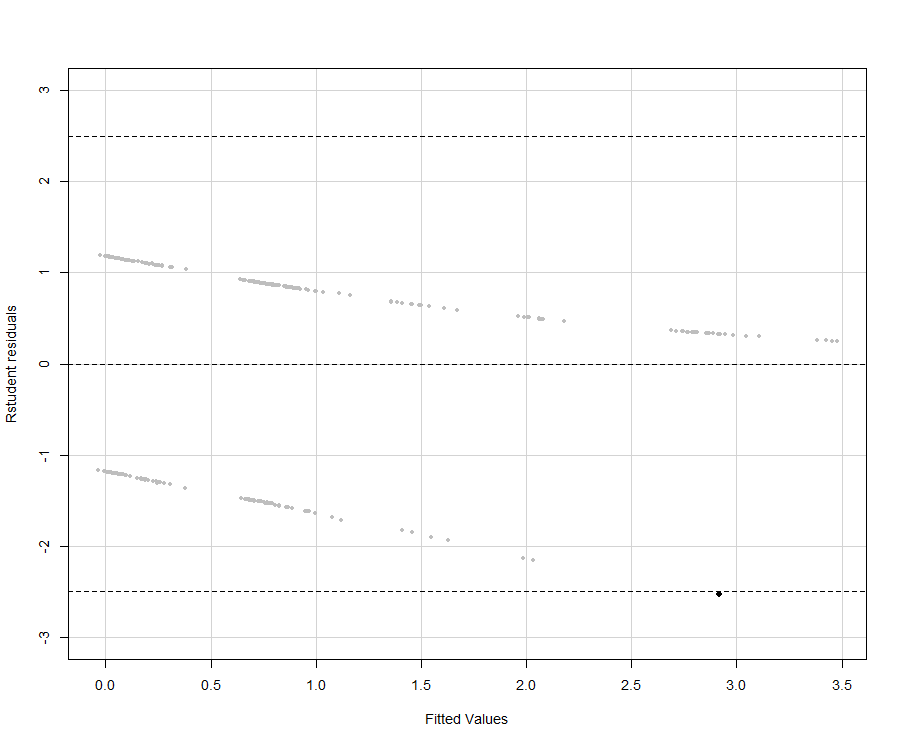


We assessed whether there were outliers by plotting studentized residuals against fitted values (Figure A2). Residuals were generally well contained within conventional bounds, with no observations exceeding ±3 (a common threshold for identifying potential outliers). The largest absolute value of the studentized residuals was -2.528 (observation 246). The influence of observations is further evaluated with influence diagnostics.

**Figure A3.** Influential observations

**
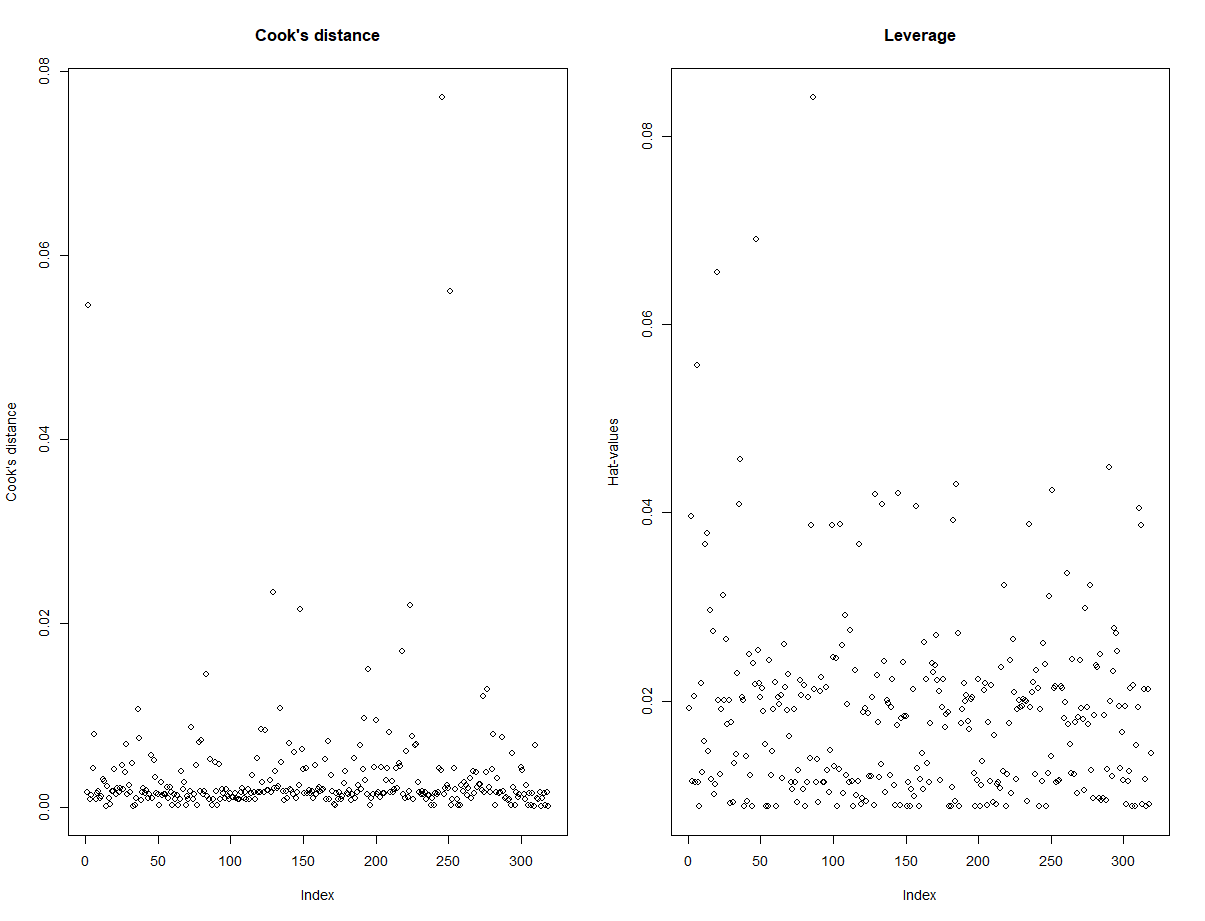
**

The majority of observations in Figure A3 have small Cook’s distances, clustered near zero. Although a small number of observations showed modestly higher Cook’s distances, none were suggestive of undue influence. Leverage values were generally low, and no observations demonstrated extreme leverage. Observation 246 did not appear influential in these diagnostic plots.

**Figure A4**. Plots of simulated, scaled residuals and predicted values


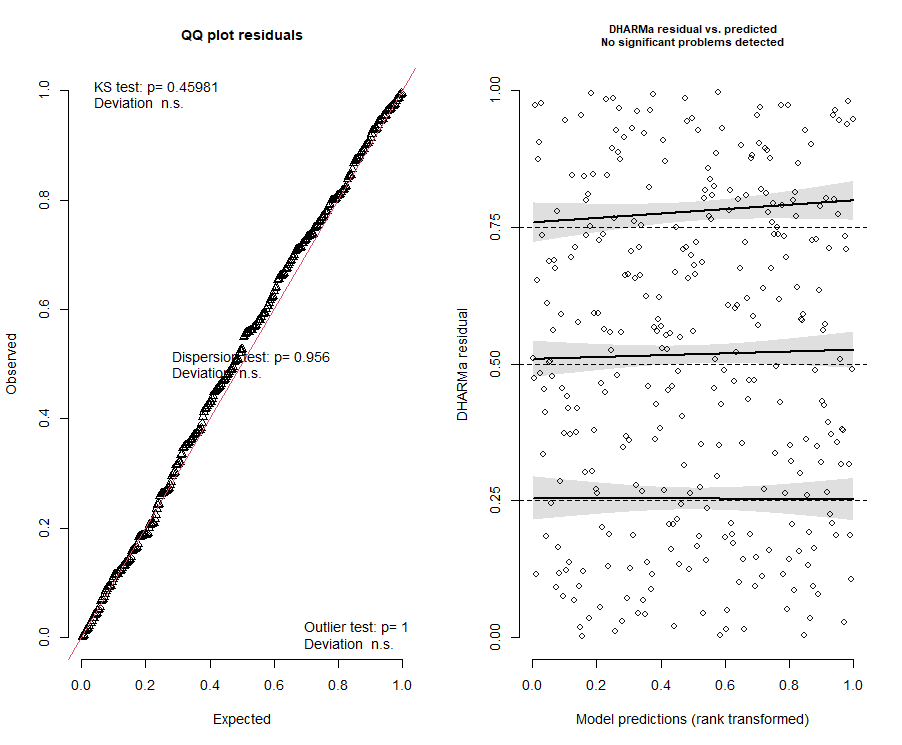


Figure A4 presents simulation-based diagnostics using DHARMa for overall model fit. Scaled residuals were approximately uniformly distributed and no significant deviations were detected in tests of distributional fit, overdispersion, or outliers. These results suggest no evidence of systematic lack of fit.

**Figure A5**. Plots of simulated, scaled residuals and each variable from the model
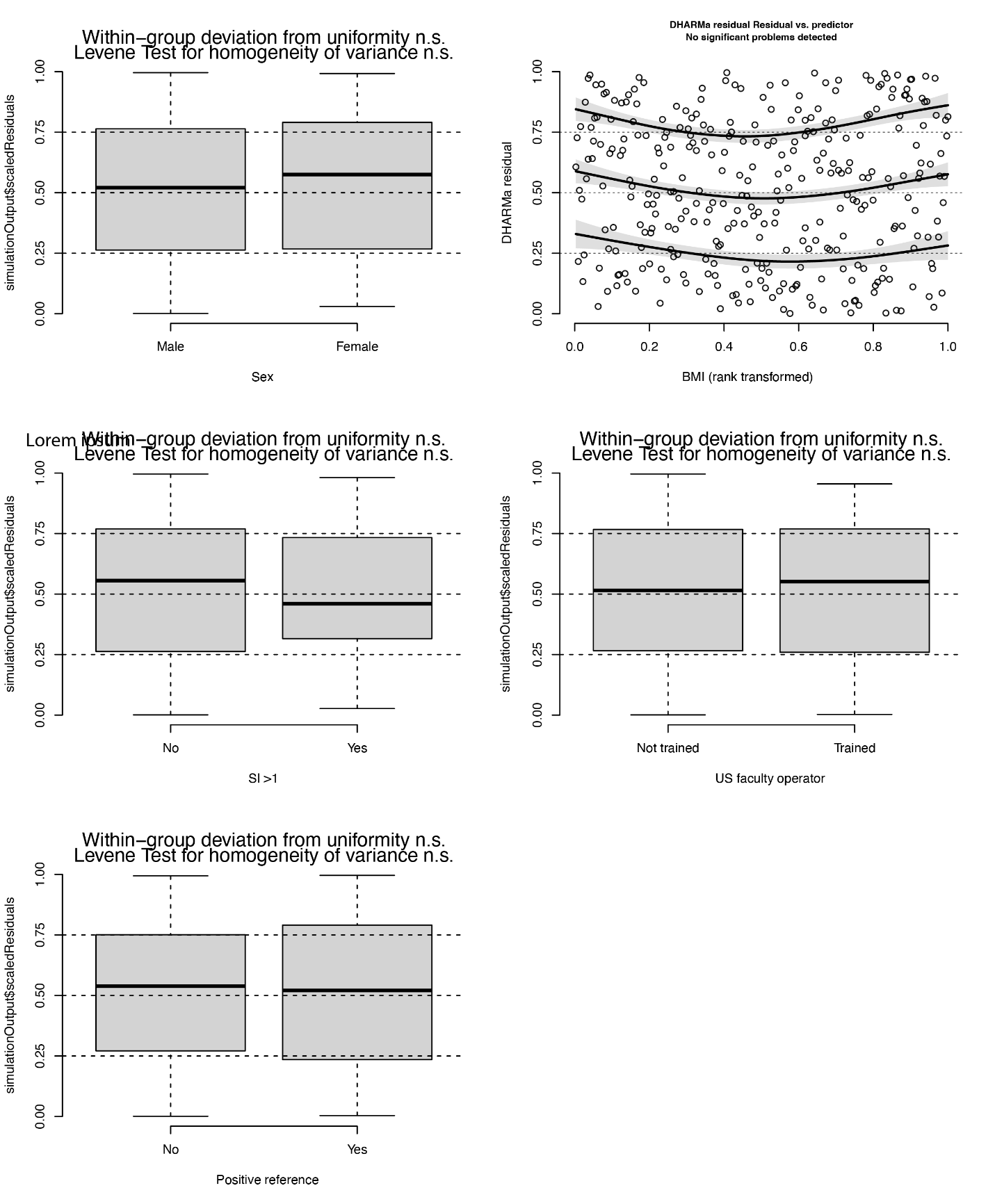


Figure A5 shows DHARMa simulated, scaled residuals plotted against each model predictor. Residuals showed no significant within-group deviations from uniformity and no evidence of heteroscedasticity. Plots of the residuals against BMI suggested mild curvature; however, this pattern was not statistically significant and did not indicate lack of fit. To further evaluate functional form, models incorporating cubic splines for BMI were compared to the model with the linear specification. Inclusion of splines did not improve model fit (likelihood ratio tests p > 0.05), thus were not used in the final model.

**Figure A6.** Calibration plot for the model of any documentation


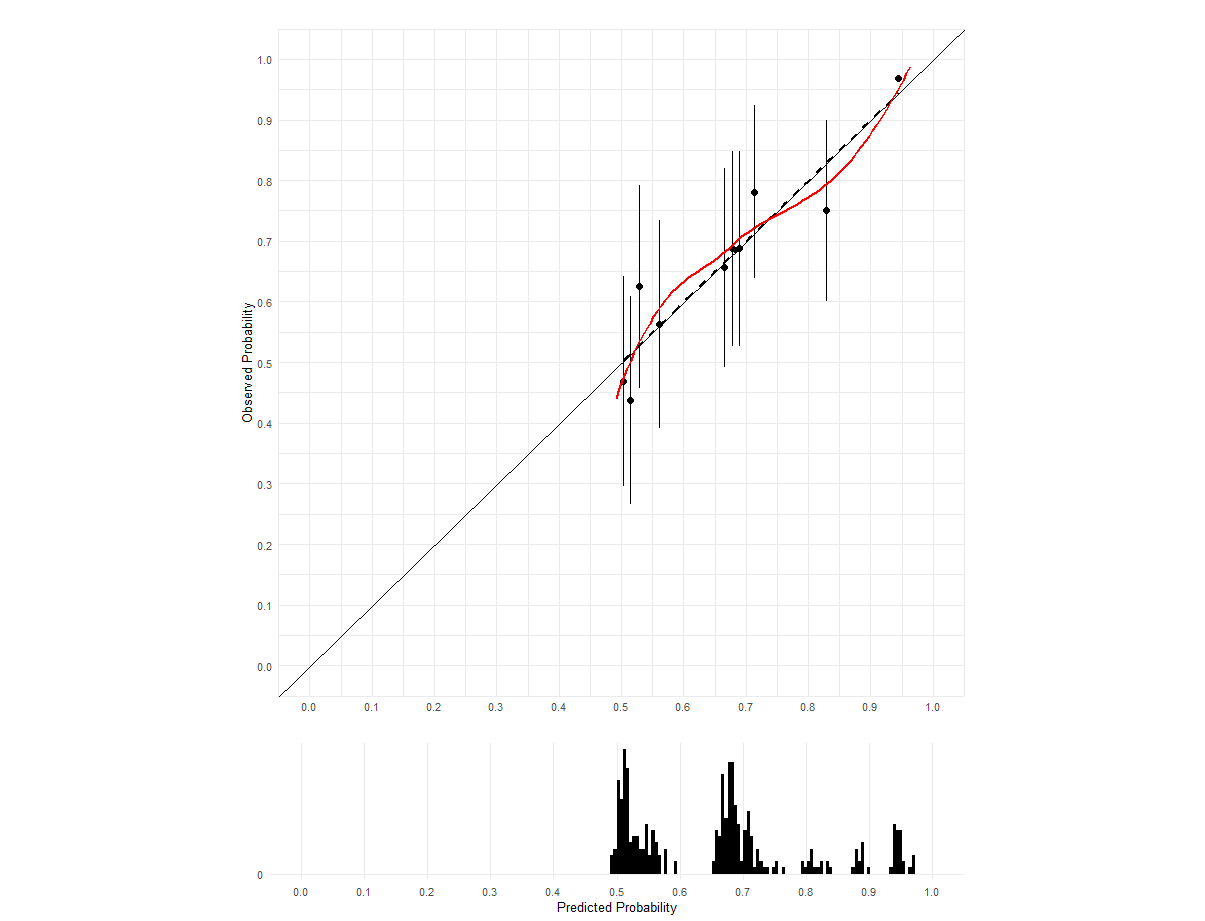


| **Table A1.** Measures of predictive model performance | |
| --- | --- |
| Measure | Estimate (95% CI)^a^ |
| Rescaled Brier score^b^ | 0.08 (0.02, 0.18) |
| Emax | 0.03 (0.00, 0.19) |
| Eavg | 0.01 (0.00, 0.04) |
| C-statistic | 0.67 (0.62, 0.74) |
| Abbreviations: CI=confidence interval, Emax=maximum absolute calibration error, Eavg=average absolute calibration error.  ^a^95% confidence intervals based on 1,000 bootstrapped samples;  ^b^Brier score was rescaled from 0 to 1 with higher values indicating better performance | |

Figure A6 shows close agreement between observed and predicted probabilities, with most points lying near the diagonal line representing ideal calibration and only minor deviations at the extremes, indicating good apparent calibration. Given the high event rate of approximately 66%, predicted probabilities were centered at higher values, with a median of 0.67 (interquartile range: 0.53, 0.71; range: 0.49, 0.97). Overall, the calibration plot was consistent with the metrics presented in Table A1, which indicate moderate discrimination (C-statistic=0.67), modest overall predictive performance (rescaled Brier score=0.08), and strong internal calibration accuracy (Eavg=0.01; Emax=0.03).

| **Table A2.** Variance inflation factor for variables included in model | |
| --- | --- |
| **Variable** | **Variance  InflationFactor** |
| SI > 1 | 1.01 |
| Positive reference | 1.03 |
| US faculty operator | 1.02 |
| Female | 1.00 |
| BMI | 1.01 |

The variance inflation factor (VIF) was estimated to be around 1 for all variables (Table A2), indicating no issues with multicollinearity among variables.

**Model diagnostics: Billing documentation**

**Figure A7.** Plots of Pearson residuals versus predictors and predicted values


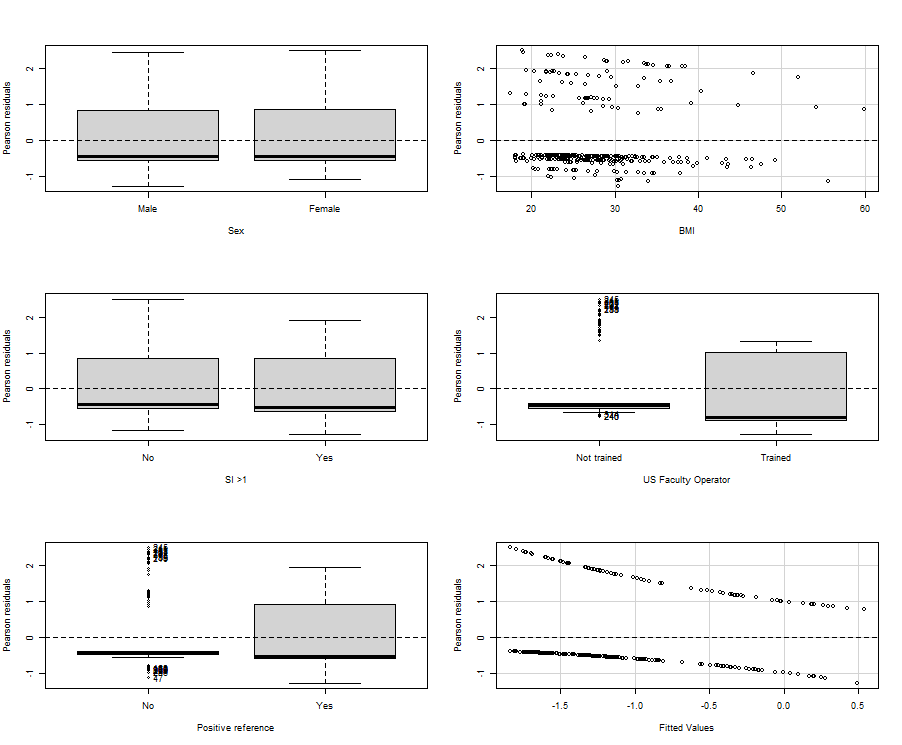


Figure A7 displays Pearson residuals plotted against individual predictors and fitted values for the secondary outcome. For sex and SI >1, residuals were centered near zero with comparable spread across the categories, suggesting no meaningful differences in model fit by these covariates. Plots for operator and positive reference demonstrate some asymmetry, characterized by a concentration of negative residuals with a wider dispersion of positive residuals. These patterns, however, are consistent with the discrete nature of the binary outcome and differences in prevalence across categories, rather than clear violations of model assumptions. Residuals plotted against BMI showed no obvious curvature or distinct patterns, although several higher positive residuals were observed at lower BMI values, along with a small number of observations at extreme BMI values (>50). Overall, there was no strong visual evidence of nonlinearity on the logit scale. The residuals versus fitted values plot does not indicate departure from linearity or lack of fit. As with the primary outcome, these plots were supplemented with simulation-based residual diagnostics and influence analyses to further investigate model adequacy.

**Figure A8.** Plot of studentized residuals vs fitted values to identify potential outliers


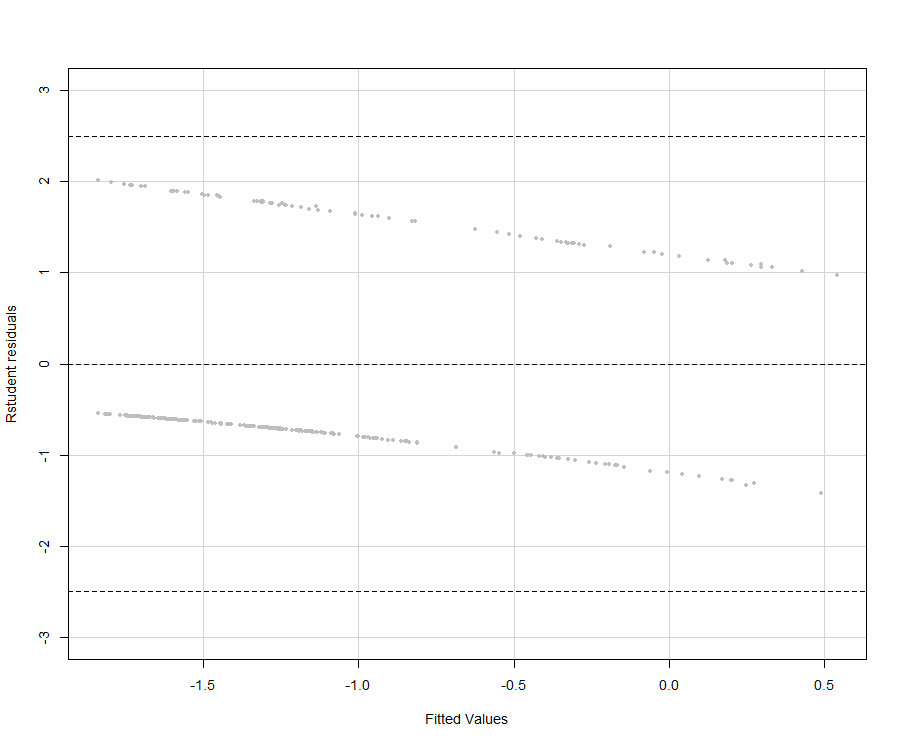


Figure A8 displays studentized residuals plotted against fitted values. Residuals were distributed around zero and all observations fell well within ±2.5. Influence diagnostics were examined to further assess the impact of individual observations.

**Figure A9.** Influential observations

**
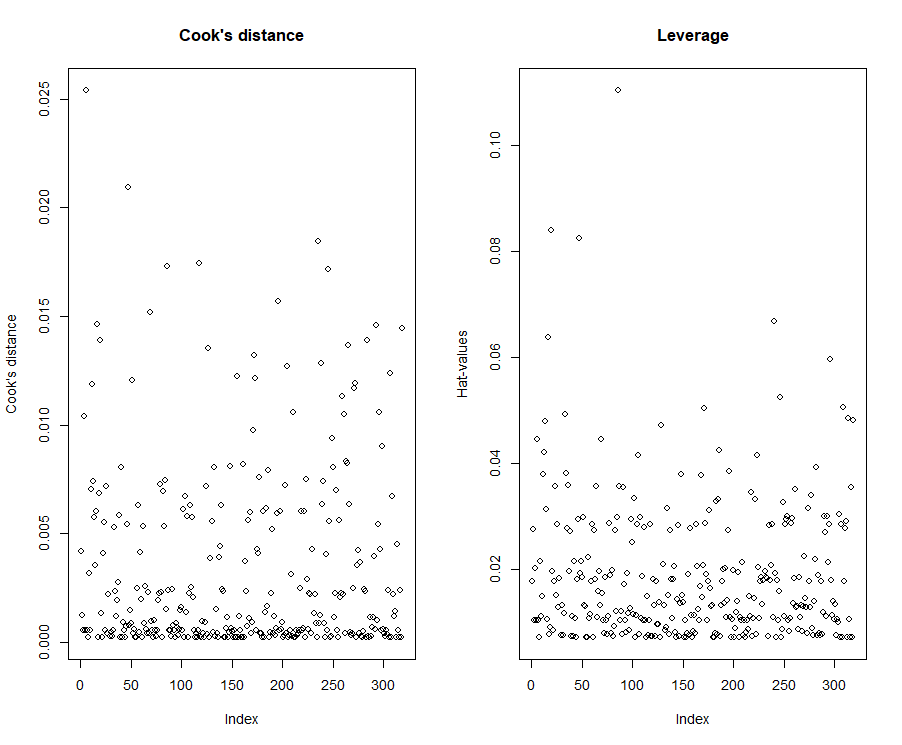
**

Figure A9 shows Cook’s distance and leverage diagnostics. All Cook’s distance values were small and below conventional thresholds, indicating no influential observations. A few observations had moderate leverage, but none corresponded to large residuals. Overall, there was no evidence of influential outliers affecting model estimates.

**Figure A10**. Plots of simulated, scaled residuals and predicted values


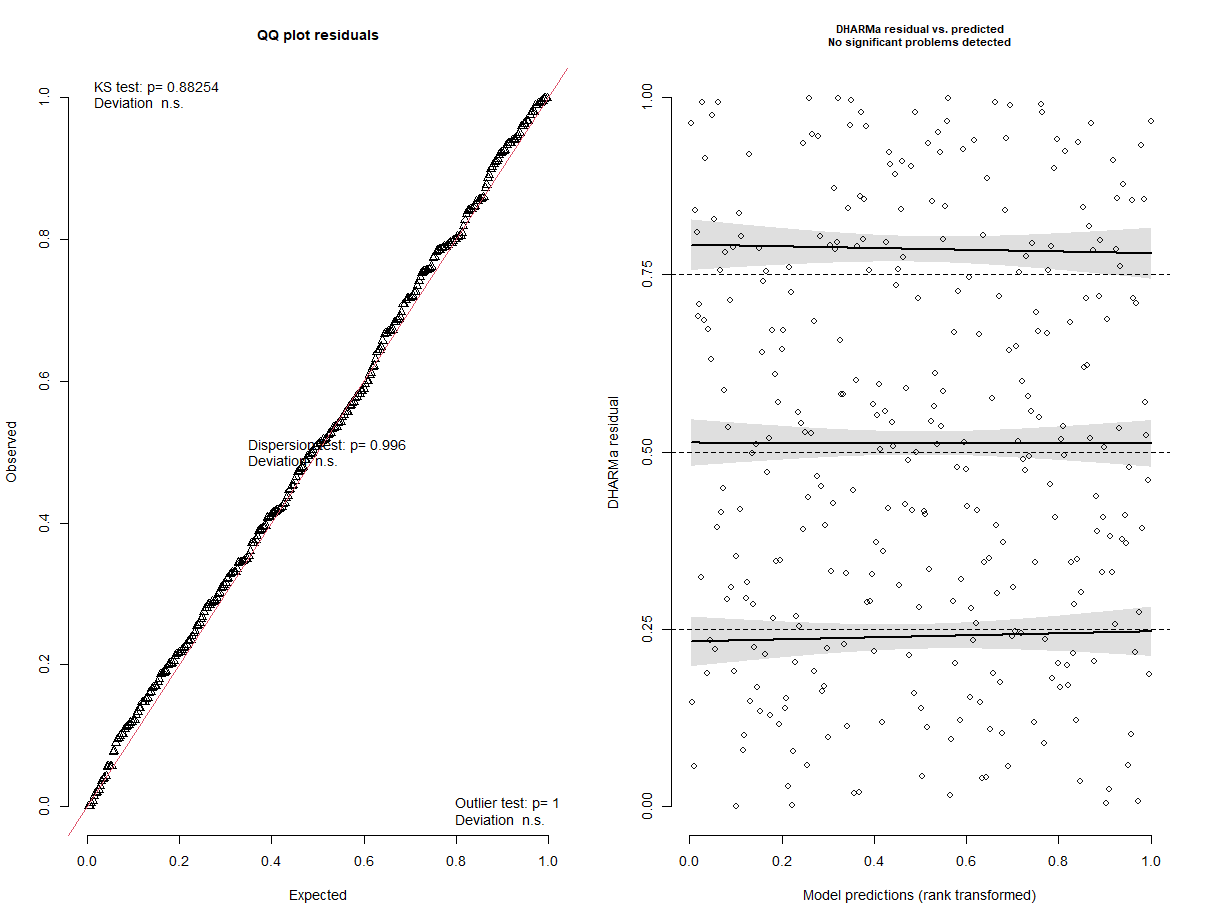


The QQ plot in Figure A10 demonstrates close agreement between observed and theoretical uniform residual distributions. Formal DHARMa tests found no evidence of overdispersion, zero-inflation, or outliers. Simulated residuals plotted against predicted values showed no systematic patterns, supporting adequate model fit.

**Figure A11**. Plots of simulated, scaled residuals and each variable from the model
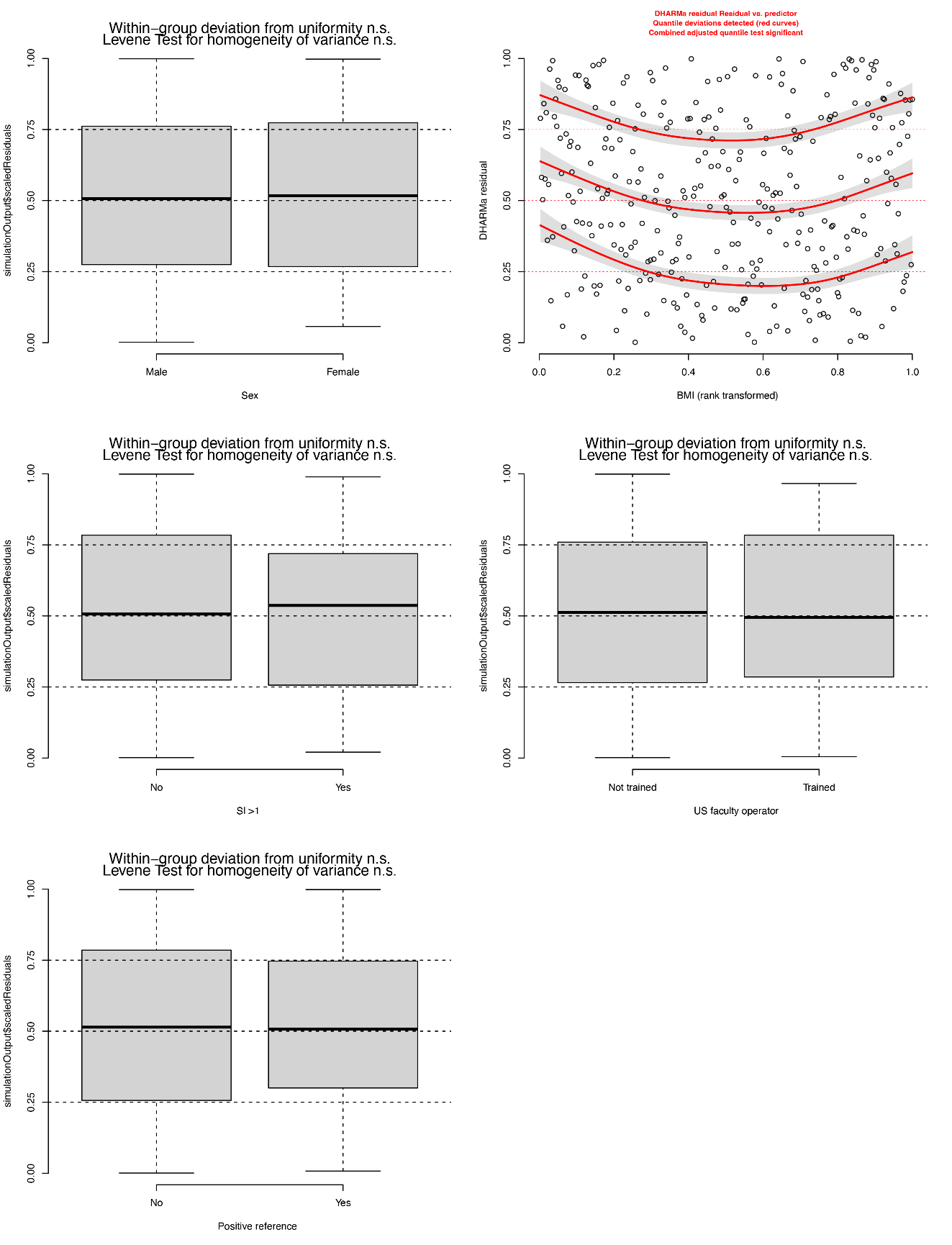


Residual distributions were approximately uniform within levels of each predictor (Figure A11). Although residuals showed mild dependence on BMI, inclusion of flexible spline terms and interaction effects did not improve model fit based on likelihood ratio tests. Additionally, diagnostics from simulated residuals for dispersion, uniformity, and residuals-versus-predicted values (Figure A10) were satisfactory. Consequently, BMI was retained as a linear term.

**Figure A12.** Calibration plot for the model of any documentation


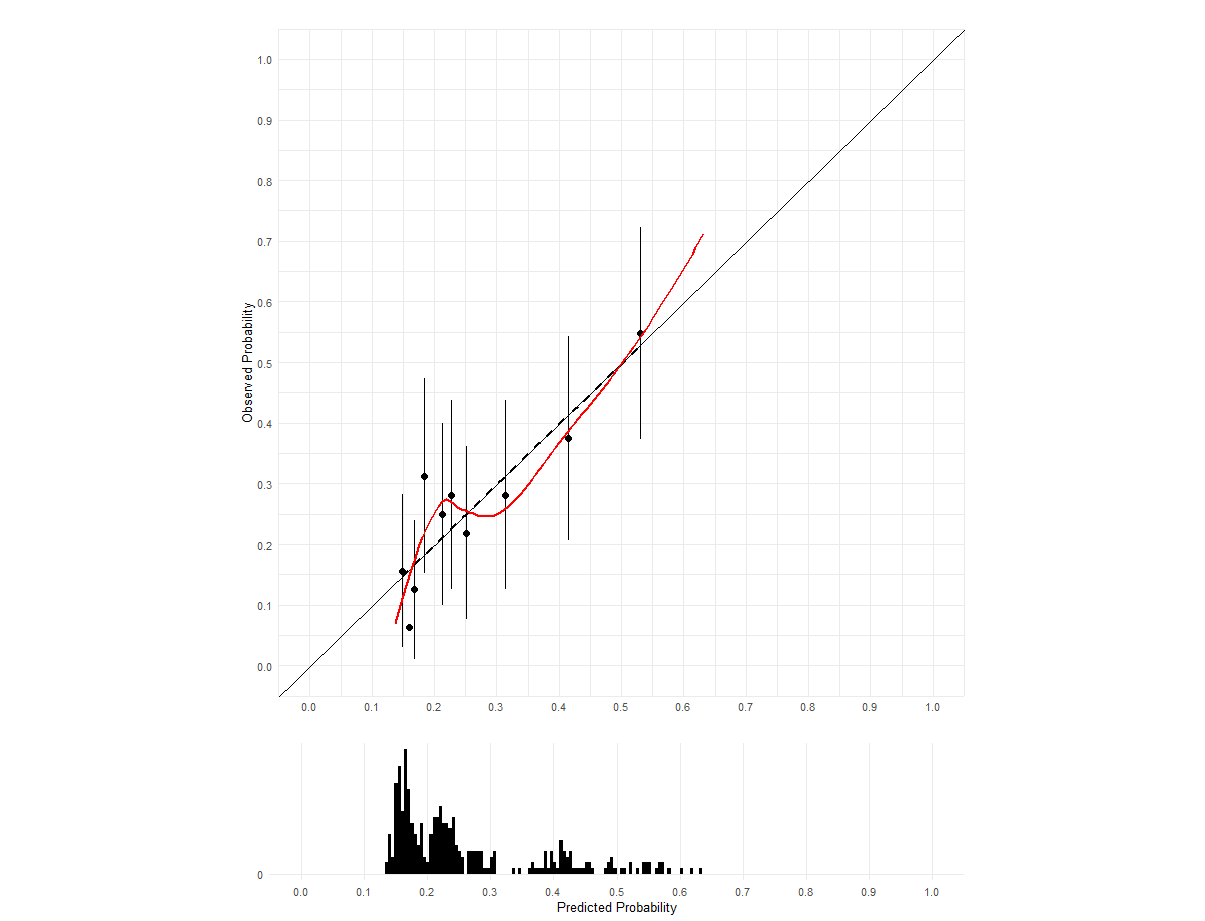


| **Table A3.** Measures of model performance | |
| --- | --- |
| Measure | Estimate (95% CI)^a^ |
| Rescaled Brier score^b^ | 0.08 (-0.02, 0.23) |
| Emax | 0.05 (0.03, 0.29) |
| Eavg | 0.02 (0.01, 0.05) |
| C-statistic | 0.66 (0.60, 0.74) |
| Abbreviations: CI=confidence interval, Emax=maximum absolute calibration error, Eavg=average absolute calibration error.  ^a^95% confidence intervals based on 1,000 bootstrapped samples;  ^b^Brier score was rescaled from 0 to 1 with higher values indicating better performance | |

Figure A12 shows good agreement between observed and predicted probabilities, with the loess curve following the diagonal reference line. Minor deviations are evident in the mid-range of predicted risk and uncertainty increase at higher predicted probabilities where data are sparse. These patterns are consistent with sample variation rather than systematic miscalibration.

Table A3 summarizes model performance. The rescaled Brier score indicates modest improvement in probabilistic accuracy relative to a null model. Calibration error was low as measured by average absolute calibration (Eavg=0.03) and maximum absolute (Emax=0.05) errors; discrimination was modest (C-statistic – 0.66). Overall, these results indicate moderate calibration and stable performance, consistent with the model’s purpose as a risk factor analysis.

| **Table A4.** Variance inflation factor for variables included in model | |
| --- | --- |
| **Variable** | **Variance  InflationFactor** |
| SI > 1 | 1.04 |
| Positive reference | 1.07 |
| US faculty operator | 1.04 |
| Female | 1.00 |
| BMI | 1.01 |

VIF was <2 for all variables (Table A4), indicating no evidence of multicollinearity.

**References**

Zhang Z. Residuals and regression diagnostics: focusing on logistic regression. *Ann Transl Med*. 2016 May;4(10):195. doi: 10.21037/atm.2016.03.36.

Dunn PK, Smyth GK. Randomized quantile residuals. *Journal of Computational and graphical statistics*. 1996 Sep 1;5(3):236-44.

Gelman A, Hill J. *Data analysis using regression and multilevel/hierarchical models*. Cambridge university press; 2007.

Hartig, F. (2024) DHARMa: Residual diagnostics for hierarchical (multi-level/mixed) regression models (Version 0.4.7) [R package]. Available at: <https://cran.r-project.org/package=DHARMa>.

1. **Detail of methods used for sensitivity analyses**

Odds ratios in logistic regression are often estimated using maximum-likelihood (ML) estimation as used in our study. When some combinations of the outcome and independent variables have sparse representation (i.e., few study participants have the combination), ML estimation may bias odds ratios away from the null and produce wide confidence intervals. Greenland and colleagues refer to this as “sparse data bias”; they recommend repeating analyses with a method more robust to bias and comparing results to those of ML (Greenland et al., 2016). Bayesian logistic regression is a known way to reduce bias in model parameters by shrinking overestimated ML estimates using mild penalization and log-F priors are a popular and natural conjugate-prior family for logistic regression (Greenland, 2007; Discacciati et al., 2015). Further, a data augmentation approach to implement the penalization implied by the prior distribution is an easy-to-implement alternative to Markov chain Monte Carlo for these Bayesian models and avoids complex convergence issues, which can be problematic with sparse data (Sullivan and Greenland, 2013; Discacciati et al., 2015). The log-F(1, 1) prior is frequently used in medical research and recommended over other priors (Greenland and Mansournia, 2015; Rahman et al., 2017).

To assess sparse data bias, we repeated univariate and multivariable analyses for our primary outcome using ML, Bayesian data augmentation with log-F(1,1) prior where the mode of the prior distribution – equal to the mean in this case because of symmetry – is υ=0 (zero is frequently used to avoid criticism for arbitrary specification in this type of Bayesian analysis), and Bayesian data augmentation with log-F(1,1) prior where the prior mean is estimated empirically (using Estimator 1 from Gosho et al. 2025, which the authors preferred). We include both versions of log-F(1, 1) prior because, while the mean υ=0 can reduce the sparse data bias when the null hypothesis is true (OR=1), Gosho and colleagues (2023) suggest that it may excessively shrink estimates of the OR under the alternative hypothesis (OR>1). Estimating the prior mean empirically both removes the arbitrariness of a subjective choice for the prior mean and also avoids potential overcorrection of the OR under the alternative hypothesis, so it provides a meaningful comparison. We report odds ratios and corresponding 95% confidence intervals for shock index, the variable that raised our suspicion of being impacted by sparse data bias, from these models.

**References**

Discacciati A, Orsini N, Greenland S. Approximate Bayesian logistic regression via penalized likelihood by data augmentation. *The Stata Journal*. 2015 Oct;15(3):712-36.

Gosho M, Ohigashi T, Nagashima K, Ito Y, Maruo K. Bias in odds ratios from logistic regression methods with sparse data sets. *Journal of epidemiology*. 2023 Jun 5;33(6):265-75.

Gosho M, Ishii R, Nagashima K, Noma H, Maruo K. Determining the prior mean in Bayesian logistic regression with sparse data: a nonarbitrary approach. Journal of the Royal Statistical Society Series C: *Applied Statistics*. 2025 Jan;74(1):126-41.

Greenland S. Prior data for non‐normal priors. *Statistics in medicine*. 2007 Aug 30;26(19):3578-90.

Greenland S, Mansournia MA. Penalization, bias reduction, and default priors in logistic and related categorical and survival regressions. *Statistics in medicine*. 2015 Oct 15;34(23):3133-43.

Greenland S, Mansournia MA, Altman DG. Sparse data bias: a problem hiding in plain sight. *BMJ*. 2016 Apr 27;352.

Rahman MS, Sultana M. Performance of Firth-and logF-type penalized methods in risk prediction for small or sparse binary data. *BMC medical research methodology*. 2017 Dec;17:1-5.

Sullivan SG, Greenland S. Bayesian regression in SAS software. *International journal of epidemiology*. 2013 Feb 1;42(1):308-17.

| **Table S1**. Unadjusted and adjusted odds ratios and 95% confidence intervals for the association of any documentation and shock index for different estimation methods | | |
| --- | --- | --- |
|  | **Univariate**  **Model** | **Multivariable**  **Model** |
| **Estimation method** | **OR (95% CI)** | **aOR (95% CI)** |
| ML estimation^a^ | 8.01 (2.81, 33.71) | 7.46 (2.57, 31.66] |
| Log F(1,1) with υ=0^b^ | 7.01 (2.61, 26.15) | 6.52 (2.39, 24.55) |
| Log F(1,1) with empirically estimated υ^c^ | 8.00 (2.90, 30.57) | 7.45 (2.66, 28.66) |
| Abbreviations: ML=maximum likelihood, OR=odds ratio, aOR=adjusted odds ratio, CI=confidence interval. ^a^Estimate from maximum likelihood estimation ^b^Bayesian data augmentation with log F(1,1) based on υ=0 ^c^Bayesian data augmentation with log F(1,1) based on empirically estimated υ | | |

**Standards for Point-of-care Ultrasound Research Reporting Checklist**

| **Section** | **Items and Subitems** | **Reported on page #** |
| --- | --- | --- |
| 1. Technical Variables & Machine Settings | The ultrasound hardware used in the study. | 4 |
|  | Machine manufacturer(s) | 4 |
|  | Machine model | 4 |
|  | Transducer type | 4 |
|  | Transducer frequency range | n/a |
|  | The exam settings used in the study. | 4 |
|  | Factory preset(s) used | n/a |
|  | Software package(s) (e.g. AI, image guidance, and/or measurement packages) | n/a |
|  | Use of Doppler mode(s). | N/a |
|  | Type of Doppler (power, color, spectral [e.g. PWD/CWD], tissue) |  |
| 2. Ultrasound Exam | The acquired ultrasound exam protocol. | 3 |
|  | Describe the rationale for selecting a particular exam protocol | 3 |
|  | Specify if the exam protocol has been described previously (e.g. novel protocol, protocol described in case reports, endorsed by professional society) | 3 |
|  | Specify all the view(s) acquired | 3 |
|  | Specify the minimum view(s) required for inclusion in analysis | 3 |
|  | If some views were acquired, but not required for analysis, specify reasons for not including those views in the analysis | 3 |
|  | The setting and set-up of the ultrasound exam. | 4 |
|  | The location of the exam (e.g. ED, ward, private room) | 4 |
|  | The position of the patient (e.g. prone, sitting, supine) | n/a |
| 3. Participant/  Subject | Variables that are specific to the subject(s) baseline or demographic characteristics AND may affect ultrasound findings. | 6 |
|  | Age | T1 |
|  | Sex | T1 |
|  | Gender | T1 |
|  | BMI | T1 |
|  | Variables that are specific to the subject(s) illness severity AND may affect ultrasound findings. | T1 |
| 4. Operator | Variables related to the operators' medical training. | 4,5, 6 |
|  | Level of medical training (e.g. PA/NP, research associate, medical student, resident with PGY level, fellow, attending, etc) | 4-5 |
|  | Level of training in ultrasound specifically (e.g. ultrasound fellowship, course completion, RDMS, EUFAC FPD or other training of the sonologist) | 4-5 |
|  | Variables related to the operators' prior experience with ultrasound. | 5 |
|  | The number of STUDY-SPECIFIC ultrasound exams ever performed by the operator (may be reported as a range e.g. 25-50, 50-100, >100 exams) | Not reported due to variability |
|  | Variables related to the operator's training on the study ultrasound protocol/exam. | 5 |
|  | Specify whether study-specific training occurred | 5 |
|  | Describe the training protocol structure (e.g. didactics, asynchronous learning, hands-on, image review, washout period) | 5 |
|  | Specify the total length of time spent on training for the study protocol | 5 |
|  | Specify whether a certain proportion of hands-on exams performed in training had to be normal or abnormal | n/a |
|  | Define a standard to assess proficiency in the study ultrasound protocol/exam needed to enroll subjects in the study (e.g. number of scans, inter-rater reliability between operator and expert, proportion of interpretable exams) | n/a |
|  | Specify the background of the trainer who provided training of the operators | 5 |
|  | Variables related to the operators' knowledge and/or blinding of the subjects' clinical presentation. | 5, not blinded |
|  | Specify if the operator is blinded to the subjects' clinical history | 5 |
|  | Specify if the operator is blinded to the subjects' clinical test results (e.g. labs and imaging) | 5 |
|  | Specify the timing when the operator performed the ultrasound with respect to the overall clinical care timeline (e.g. after history, but before CT or other confirmatory testing) | 5 |
|  | Variables related to the operators' interaction with the clinical team taking care of the subject. | 5 |
|  | Specify whether the ultrasound operator is a member of the clinical team taking care of the subject | 5 |
|  | Specify whether the ultrasound operator is communicating all ultrasound results to the clinical team taking care of the subject | n/a |
|  | If the clinical team is generally blinded to ultrasound results, specify whether the ultrasound operator is communicating some critical or incidental ultrasound results to the clinical team (e.g. unexpected finding of tamponade in a study of patients with heart failure). | n/a |
|  | Describe how the ultrasound operator and ultrasound exam may be impacting clinical management | 5 |
| 5. Data Analysis and Interpretation | Assessment of image quality must be described in sufficient detail to allow replication. | n/a |
|  | Specify the qualifications of the image quality reviewer(s) |  |
|  | Specify whether the image quality reviewer(s) are involved in other portions of the study |  |
|  | Specify the scale by which image quality is assessed |  |
|  | Variables related to the interpretation of ultrasound results used in the study analysis. | 5-6 (and supplement) |
|  | Specify the qualifications of the reviewers who are interpreting the POCUS results | 5-6 (and supplement) |
|  | Specify whether the operator provides interpretation of the POCUS results | 5-6 (and supplement) |
|  | Specify the number of reviewers interpreting the POCUS results | n/a |
|  | Specify how disagreements between reviewer interpretations are resolved | n/a |
|  | Provide a measure of inter-rater reliability between the people interpreting the POCUS results | n/a |
|  | If more than one aspect of the POCUS is being interpreted, a range of inter-rater reliability measures should be reported (e.g. a cardiac ultrasound study should report separate kappa values for interpretation of LV function, RV size, and RV function) | n/a |
|  | When more than one reviewer provides an interpretation, describe which interpretation is used for the final analysis | n/a |
|  | Specify whether the reviewer(s) providing interpretation are blinded to the subjects' clinical data | n/a |
|  | When more than one reviewer provides POCUS interpretation, the methods for interpreting POCUS results and resolving disagreements must be described and adhere to a minimum standard. | n/a |
| 6. Study Specific | Authors should continue to utilize any pre-existing reporting guidelines relevant to the study type (e.g. EQUATOR network guidelines). |  |
|  | Randomized Controlled Trials: CONSORT guideline | n/a |
|  | Observational Studies: STROBE | 5 |
|  | Systematic Reviews: PRISMA | N/a |
|  | Diagnostic Studies: STARD | n/a |
|  | Prognostic Studies: TRIPOD | n/a |
|  | Case Reports: CARE (when feasible based on journal guidelines) | n/a |
|  | Studies evaluating the use of ultrasound in pediatric patients must describe ultrasound specific methods in sufficient detail to allow replication. | n/a |
|  | Specify the position of the child during the ultrasound exam |  |
|  | Specify the effect of age-range specific anatomy and physiology on the scan protocol |  |
|  | Specify whether parental consent and/or patient assent was obtained |  |
|  | Studies evaluating the use of ultrasound for procedural guidance must describe ultrasound specific methods in sufficient detail to allow replication. | n/a |
|  | Whenever possible, a comparator group should be included in studies evaluating the use of ultrasound for procedural guidance |  |
|  | Specify whether ultrasound was used in a dynamic (simultaneous imaging and procedure performance) or static (separate steps for imaging and procedure performance) manner |  |
|  | Clearly specify and justify the outcome measure in a manner that allows replication |  |
|  | If a simulator or phantom was used, describe this in sufficient detail to allow replication |  |
|  | Studies evaluating the use of POCUS in LRS warrant specific reporting considerations. | n/a |
|  | POCUS studies in LRS hold value even if complete reporting is not possible due to limitation of resources |  |
|  | When possible, POCUS studies in LRS should describe the relevant environment in sufficient detail |  |
|  | When possible, POCUS studies in LRS should describe the availability of relevant comprehensive/radiology performed ultrasound imaging |  |
|  | When possible, POCUS studies in LRS should describe the availability of relevant non-ultrasound imaging services |  |

AI, artificial intelligence; PWD, pulsed wave doppler; CWD, continuous wave doppler; BMI, body mass index; ED, Emergency Department; PA, physician assistant; NP, nurse practitioner; RDMS, registered diagnostic medical sonographer; PGY, post-graduate year; EUFAC, Emergency Ultrasound Fellowship Accreditation Council; FPD, focused practice designation; POCUS, point-of-care ultrasound; LV, left ventricle; RV, right ventricle; LRS, limited resource settings

**STROBE - STrengthening the Reporting of OBservational studies in Epidemiology checklist**

|  | Item No | Recommendation | Page No |
| --- | --- | --- | --- |
| **Title and abstract** | 1 | (*a*) Indicate the study’s design with a commonly used term in the title or the abstract | Abstract |
|  |  | (*b*) Provide in the abstract an informative and balanced summary of what was done and what was found | Abstract |
| Introduction | | | |
| Background/rationale | 2 | Explain the scientific background and rationale for the investigation being reported | 3 |
| Objectives | 3 | State specific objectives, including any prespecified hypotheses | 4 |
| Methods | | | |
| Study design | 4 | Present key elements of study design early in the paper | 4 |
| Setting | 5 | Describe the setting, locations, and relevant dates, including periods of recruitment, exposure, follow-up, and data collection | 4 |
| Participants | 6 | (*a*) Give the eligibility criteria, and the sources and methods of selection of participants. Describe methods of follow-up | 5 |
|  |  | (*b*) For matched studies, give matching criteria and number of exposed and unexposed | n/a |
| Variables | 7 | Clearly define all outcomes, exposures, predictors, potential confounders, and effect modifiers. Give diagnostic criteria, if applicable | 5-6 |
| Data sources/ measurement | 8* | For each variable of interest, give sources of data and details of methods of assessment (measurement). Describe comparability of assessment methods if there is more than one group | 5-6 |
| Bias | 9 | Describe any efforts to address potential sources of bias | 5 (consecutive enrollment) |
| Study size | 10 | Explain how the study size was arrived at | 5 |
| Quantitative variables | 11 | Explain how quantitative variables were handled in the analyses. If applicable, describe which groupings were chosen and why | 7 |
| Statistical methods | 12 | (*a*) Describe all statistical methods, including those used to control for confounding |  |
|  |  | (*b*) Describe any methods used to examine subgroups and interactions | 7 and Supplement |
|  |  | (*c*) Explain how missing data were addressed |  |
|  |  | (*d*) If applicable, explain how loss to follow-up was addressed |  |
|  |  | (*e*) Describe any sensitivity analyses |  |
| Results | | |  |
| Participants | 13* | (a) Report numbers of individuals at each stage of study—eg numbers potentially eligible, examined for eligibility, confirmed eligible, included in the study, completing follow-up, and analysed | 8 |
|  |  | (b) Give reasons for non-participation at each stage | n/a |
|  |  | (c) Consider use of a flow diagram | Fig. 1 |
| Descriptive data | 14* | (a) Give characteristics of study participants (eg demographic, clinical, social) and information on exposures and potential confounders | Table 1 |
|  |  | (b) Indicate number of participants with missing data for each variable of interest | Table 1 |
|  |  | (c) Summarise follow-up time (eg, average and total amount) | N/a |
| Outcome data | 15* | Report numbers of outcome events or summary measures over time | 8-11  Fig 1. |

| Main results | 16 | (*a*) Give unadjusted estimates and, if applicable, confounder-adjusted estimates and their precision (eg, 95% confidence interval). Make clear which confounders were adjusted for and why they were included | T3 |
| --- | --- | --- | --- |
|  |  | (*b*) Report category boundaries when continuous variables were categorized | T3 |
|  |  | (*c*) If relevant, consider translating estimates of relative risk into absolute risk for a meaningful time period | T4 |
| Other analyses | 17 | Report other analyses done—eg analyses of subgroups and interactions, and sensitivity analyses | Supp |
| Discussion | | | |
| Key results | 18 | Summarise key results with reference to study objectives | 13 |
| Limitations | 19 | Discuss limitations of the study, taking into account sources of potential bias or imprecision. Discuss both direction and magnitude of any potential bias | 14-15 |
| Interpretation | 20 | Give a cautious overall interpretation of results considering objectives, limitations, multiplicity of analyses, results from similar studies, and other relevant evidence | 15 |
| Generalisability | 21 | Discuss the generalisability (external validity) of the study results | 14-16 |
| Other information | | | |
| Funding | 22 | Give the source of funding and the role of the funders for the present study and, if applicable, for the original study on which the present article is based | Title Page |

*Give information separately for exposed and unexposed groups.

**Note:** An Explanation and Elaboration article discusses each checklist item and gives methodological background and published examples of transparent reporting. The STROBE checklist is best used in conjunction with this article (freely available on the Web sites of PLoS Medicine at http://www.plosmedicine.org/, Annals of Internal Medicine at http://www.annals.org/, and Epidemiology at http://www.epidem.com/). Information on the STROBE Initiative is available at http://www.strobe-statement.org.
